# Evaluation of Nutritional Interventions in the Care Plan for Cancer Patients: The NOA Project

**DOI:** 10.3390/nu15020292

**Published:** 2023-01-06

**Authors:** Pedro Pablo García-Luna, Juana M. Rabat Restrepo, Marta Muñoz-Ayllón, Milagros de la Calle Gil, Pablo Remón, Francisco José Sánchez-Torralvo, Jerónimo Pachón, Juan J. García-González, Teresa García-Manrique, Javier Salvador-Bofill, David Vicente, Gabriel Olveira

**Affiliations:** 1 Unidad de Nutrición Clínica y Dietética, U. G. de Endocrinología y Nutrición, Hospital Universitario Virgen del Rocío, 41013 Sevilla, Spain; 2 Unidad de Nutrición Clínica y Dietética, S. de Endocrinología y Nutrición, Hospital Universitario Virgen Macarena, Universidad de Sevilla, 41009 Sevilla, Spain; 3 Unidad Intercentros de Oncología Médica, Hospitales Universitarios Regional y Virgen de la Victoria, 29010 Málaga, Spain; 4 Unidad de Gestión Clínica de Oncología Integral, Hospital Universitario Virgen del Rocío, 41013 Sevilla, Spain; 5 U.G.C. de Endocrinología y Nutrición, Hospital Regional Universitario de Málaga, Universidad de Málaga, 29010 Málaga, Spain; 6 Servicio de Endocrinología y Nutrición, Hospital Universitario Virgen Macarena, 41009 Sevilla, Spain; 7 Unidad de Oncología, Hospital Virgen de la Macarena, 41009 Sevilla, Spain; 8 U.G.C. de Endocrinología y Nutrición, Hospital Regional Universitario de Málaga, Universidad de Málaga, Instituto de Investigación Biomédica de Málaga-Plataforma Bionand, Centro de Investigación Biomédica en Red de Diabetes y Enfermedades Metabólicas Asociadas (CIBERDEM), 29010 Málaga, Spain

**Keywords:** nutritional status, neoplasms, nutritional assessment, nutritional support, malnutrition

## Abstract

The NOA (Oncological Nutrition in Andalusia) project analyses the degree of integration and areas of improvement in implementing nutritional support in the care plans of cancer patients in Andalusia. The aim was to analyse nutritional interventions for better care of cancer patients and for the improvement of the management of malnutrition in cancer. A prospective evaluation of the implementation of two areas of improvement in nutrition was conducted in three hospitals. Data were collected from each hospital over a six-month period using an online platform. A standardised care plan was designed for hospitals in Andalusia, in which proposed improvements were devised and prioritised, selecting nutritional screening in oncology services and the participation of the Nutrition Support Team (NST) on the tumour boards, as well as the assessment of the patients presented at these sessions. Our results indicated an increase in the number of medical records with nutritional evaluation results six months later, regardless of the type of tumour or hospitalisation; and there was greater participation of the NST on the tumour boards, mainly for head and neck and oesophagogastric cases. Solutions for improvement have been pinpointed and implemented that have positively impacted the nutritional care plan in the course of oncological disease.

## 1. Introduction

Malnutrition is a common medical condition in cancer patients [1,2]. It has negative consequences on the course of the disease in terms of quality of life and life expectancy [3,4,5,6,7], as well as on the use of healthcare resources associated with treatment [8,9].

Nutritional guidelines, scientific societies [10,11], and the European Parliament, through its Europe’s Beating Cancer Plan [12], recommend assessing the risk of malnutrition in all cancer patients at the time of diagnosis and then periodically during treatment. A complete nutritional assessment and the implementation of a personalised intervention where necessary is also recommended [13]. In addition, this plan recommends integrated collaborative care by the various professionals treating cancer patients and, consequently, the preparation of integrated health plans for nutrition/oncology. Cancer-related nutritional intervention aims to identify, prevent, and treat malnutrition through nutritional dietary counselling, with or without oral nutritional supplements or enteral or parenteral nutrition, as well as to address metabolic and nutritional changes affecting patient recovery and survival [14].

Although early nutritional assessment and intervention have been shown to positively impact the prognosis of the tumour process [15,16], these procedures are not yet part of standard clinical practice in most healthcare centres [17]. This leads to a high proportion of cancer patients with unidentified malnutrition or risk of malnutrition that is not treated early enough [18]. Furthermore, nutritional support is not generally included in oncological care protocols, and there are striking differences between hospitals and territories [19]. Thus, despite its relevance, malnutrition is still an understudied topic in cancer patients.

The NOA project aims to analyse current nutritional interventions at different phases of the cancer plan to establish a consensus on the best integrated care plan, taking into account the judgment and needs of end users, including the treating health professionals and the cancer patients subject to these assessments. Phase 1 of the project, which analysed the nutritional and oncological process in eight sites in the autonomous community of Andalusia, showed that nutritional screening is not routinely performed, nor is there a universal consensus on the nutritional intervention and action protocols to be implemented, with geographical variations and differences between the healthcare centres analysed. During this phase, opportunities for improvement within the identified care plan were also described and prioritised [20].

Phases 2 and 3 of the NOA project seek to identify proposed improvements in response to the opportunities identified in phase 1 and to evaluate the viability in clinical practice of those with higher impact and feasibility values.

## 2. Materials and Methods

### 2.1. Participants

The study, in its initial phase already published [20], involved healthcare professionals from different medical specialities at eight different centres, including hospitals and primary care services, across five provinces in the autonomous community of Andalusia. Phase 3 was conducted at three of the sites involved in the project: Hospital Virgen del Rocío and Hospital Virgen Macarena, both of which are in Seville, and Hospital Regional Universitario de Málaga, where an assessment was made on the success of the implementation of the two improvement proposals in routine clinical practice with the highest impact and feasibility values, to reduce the amount of lost data due to the lack of implementation. All participants were selected according to experience in both clinical nutrition and oncology. The names of the investigators and their affiliations are listed in Supplementary Table S1. During phase 2 of the project, the Spanish Association Against Cancer (AECC), the Andalusian School of Public Health (AESP), and the Cancer Care Foundation (CUDECA) participated to incorporate the viewpoints of the affected patients and their relatives. Likewise, the study included the director of the Andalusian Oncology Plan [21] and the head of the Support Process for Clinical Nutrition and Dietetics of the Regional Government of Andalusia (2006) [22].

### 2.2. Methodology and Project Phases

The study used design thinking techniques. As a starting point, the work team analysed the status of nutritional intervention in cancer patients at the different participating sites. Once the healthcare plans of each site were identified, a summary map was produced. This was the basis upon which opportunities for improvement were defined. Having established the areas with potential for improvement, solutions were devised and prioritised according to impact and feasibility. For each of the proposals, the main factors hindering their implementation were assessed in terms of the following: economic investment, time, legal considerations; technological, human, and material resources. In addition, using the mental mapping methodology, each mental map was described in terms of what, who, how, and when, and their complexity (basic, advanced, or excellent) was determined using a colour code. Finally, considering the ideas for improvement, a prototype of an integrated care plan (oncology and nutrition) was designed with aspects considered to be minimum essential requirements that could potentially be rolled out across all sites participating in the project. The study did not include testing of the prototype, but it did assess the outcome of implementing the two improvement ideas with the highest impact and feasibility values at some of the sites.

The NOA project was conducted in three phases. The results for phase 1 have already been published [20].

### 2.3. Indicators of Success

To assess the success of the implementation of the improvement ideas in routine clinical practice with the highest impact and feasibility values selected, different indicators were determined by the working group, which included healthcare professionals from various disciplines with experience in oncological clinical nutrition.

#### 2.3.1. In-Hospital Nutritional Screening

According to the Global Leadership Initiative on Malnutrition (GLIM) criteria [23], we undertook a two-step approach for malnutrition diagnosis, the first step involving screening to identify subjects at risk of malnutrition by the use of any validated screening tool, and the second step characterized by assessment for diagnosis and grading the severity of malnutrition.

In the autonomous community of Andalusia, the clinical nutrition and dietetic program recommend the Malnutrition Universal Screening Tool (MUST) as screening tool to identify patients at risk of malnutrition. MUST is a five-step screening tool for application in adult patients across all healthcare settings, including oncology, which has been validated in both outpatients and inpatients [24]. Among the available nutrition screening tools, MUST was selected due to its ease of use, agility, and suitability in any healthcare setting. A review comparing eight screening tools by Stratton et al. reported MUST to be one of the easiest screening tools based on the feedback by nurses, medical students, and nutritionists [24]. For instance, MUST versus the Patient-Generated Subjective Global Assessment (PG-SGA) resulted in an 86.7% sensitivity and 94.5% specificity. MUST has a high level of agreement with the PG-SGA (Kappa = 0.81; *p* < 0.05) and the highest area under the receiver operating characteristic curve (AUC ROC = 0.91). MUST has a high level of agreement with the PG-SGA in detecting outpatients undergoing chemotherapy at risk of malnutrition [25]. MUST has previously been validated with the PG-SGA in radiation oncology patients [26] and with SGA in adult oncology inpatients [27].

The outcome of the in-hospital nutritional screening (improvement idea 1.2) was assessed based on the percentage of oncology clinical histories with nutritional screening results in non-hospitalised patients and the percentage of hospitalised patients undergoing nutritional screening.

The percentage of oncology clinical histories with nutritional screening results in non-hospitalised patients was calculated as the number of clinical histories with a nutritional screening record in the clinical history divided by the total number of clinical histories evaluated (A):

$$A=\frac{\mathrm{No}.\;\mathrm{of}\;\mathrm{clinical}\;\mathrm{histories}\;\mathrm{with}\;\mathrm{nutritional}\;\mathrm{screening}\;\mathrm{results}}{\mathrm{Total}\;\mathrm{no}.\;\mathrm{of}\;\mathrm{clinical}\;\mathrm{histories}\;\mathrm{evaluated}}\times 100$$

For this, an analysis was performed in outpatient clinics and in oncology and radiation therapy day hospitals. A representative random sampling of the total clinical histories available was performed, and a minimum of 30 clinical histories per month and hospital were established to consider the indicators valid, provided the number of patients available reached this figure.

The percentage of hospitalised patients undergoing nutritional screening was calculated as the number of hospitalised patients with a nutritional screening result over the total number of hospitalised patients (B):

$$B=\frac{\mathrm{No}.\;\mathrm{of}\;\mathrm{hospitalised}\;\mathrm{patients}\;\mathrm{undergoing}\;\mathrm{nutritional}\;\mathrm{screening}}{\mathrm{Total}\;\mathrm{no}.\;\mathrm{of}\;\mathrm{hospitalised}\;\mathrm{patients}}\times 100$$

In this case, the medical records of all cancer patients hospitalised in the oncology ward or other hospital wards were analysed. For both indicators, results were collected by tumour type: head and neck, oesophagogastric, biliopancreatic, and colorectal. It was also determined in both cases that the standard would be reached once more than 50% of the clinical histories analysed had nutritional screening results.

#### 2.3.2. Nutrition Support Team Participation on Tumour Boards

The participation of the Nutrition Support Team (NST) on tumour boards (improvement idea 10.1) was assessed based on the number of board sessions in which a member of the NST participated, and on the total number of patients assessed by the NST.

The percentage of board sessions with NST participation was calculated as the number of board sessions in which a member of the NST participated over the total sessions held (C). The standard was considered to be reached when the NST participated in more than 70% of the sessions.

$$C=\frac{\mathrm{No}.\;\mathrm{of}\;\mathrm{tumour}\;\mathrm{board}\;\mathrm{sessions}\;\mathrm{with}\;\mathrm{NST}\;\mathrm{participation}}{\mathrm{Total}\;\mathrm{no}.\;\mathrm{of}\;\mathrm{tumour}\;\mathrm{board}\;\mathrm{sessions}\;\mathrm{held}}\times 100$$

The percentage of patients evaluated by the NST on the tumour board was calculated as the number of patients assessed by the NST out of the total patients presented to the board with an indication for nutritional assessment (D). For this indicator, 100% of the committee minutes and 100% of the patients referred for nutritional assessment were analysed. In this case, the standard stood at 50%.

$$D=\frac{\mathrm{No}.\;\mathrm{of}\;\mathrm{patients}\;\mathrm{assessed}\;\mathrm{by}\;\mathrm{the}\;\mathrm{NST}}{\mathrm{Total}\;\mathrm{no}.\;\mathrm{of}\;\mathrm{patients}\;\mathrm{with}\;\mathrm{indication}\;\mathrm{for}\;\mathrm{nutritional}\;\mathrm{assessment}}\times 100$$

In both cases, the selected committees were head and neck, oesophagogastric, biliopancreatic, and colorectal.

#### 2.3.3. Indicator Collection

The results for each of the success indicators were collected during the period between March and September 2021. March data were used as a reference. The investigators responsible for each of the sites logged the data on an online platform designed for this purpose. Data were collected monthly during the first quarter of the project (April 2021–June 2021) and in aggregate for the last three months (July 2021–September 2021).

## 3. Results

### 3.1. Improvement Ideas

**Figure 1:**
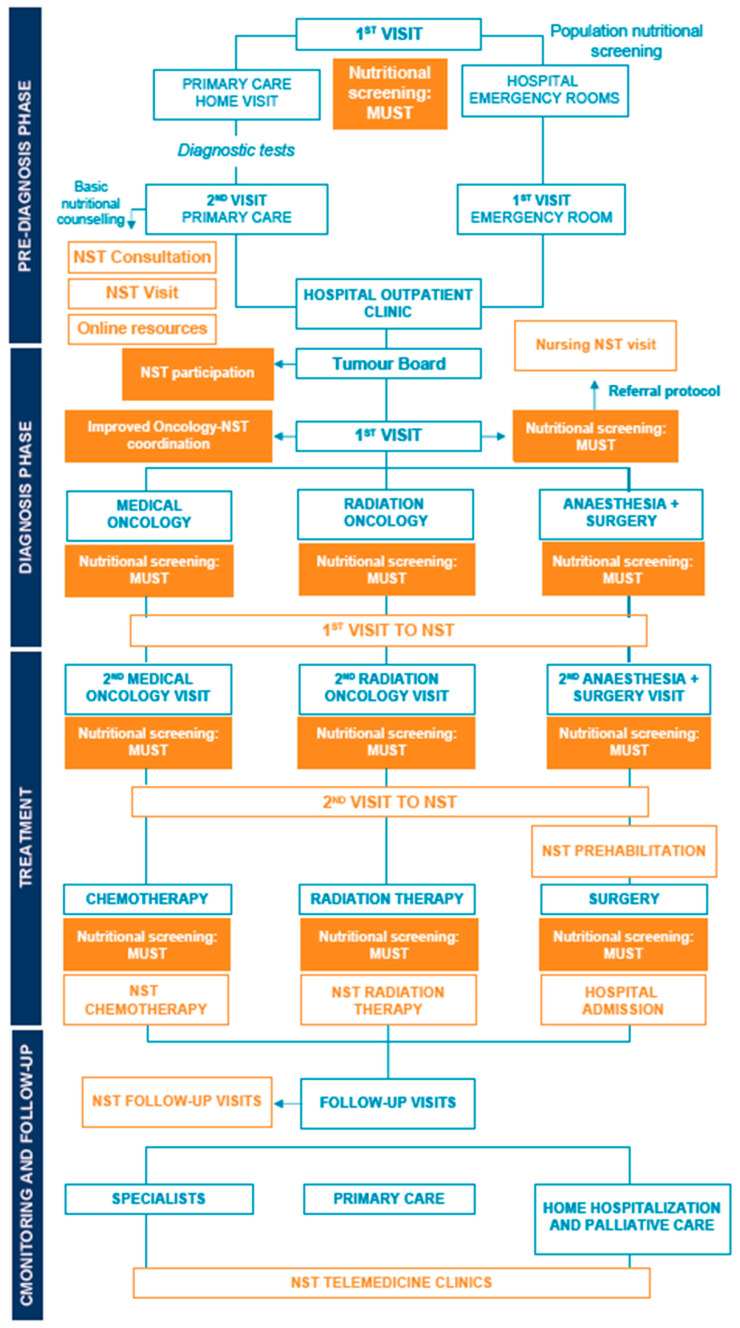
Prototype of the integrated care plan, including oncology and nutrition plans, during pre-diagnosis, diagnosis, treatment, monitoring, and follow-up phases. A summary map was produced by identifying the status of nutritional intervention in cancer patients and healthcare plans of eight sites of the autonomous community of Andalusia using a design thinking technique. This was the basis upon which the areas with potential for improvement were defined. Solutions were devised and prioritized according to impact and feasibility. Considering the ideas for improvement, a prototype of an integrated care plan (oncology and nutrition) was designed with aspects considered to be minimum essential requirements that could potentially be rolled out across all healthcare sites. Orange depicts the interventions that are part of the nutrition plan. Priority improvement ideas are shaded. MUST: Malnutrition Universal Screening Tool; NST: Nutritional Support Team.

The results presented are based on a prior analysis of the oncological and nutrition care plan in eight healthcare sites of the autonomous community of Andalusia, from which a summary map was designed (Figure 1) and in which opportunities for improvement were identified [20].

All improvement proposals made in response to the opportunities identified within the integrated care plans are shown in Table 1, together with the primary obstacles detected for their implementation and the phase in which they are implemented (pre-diagnosis, diagnosis, treatment, monitoring, and/or follow-up).

**Figure 2:**
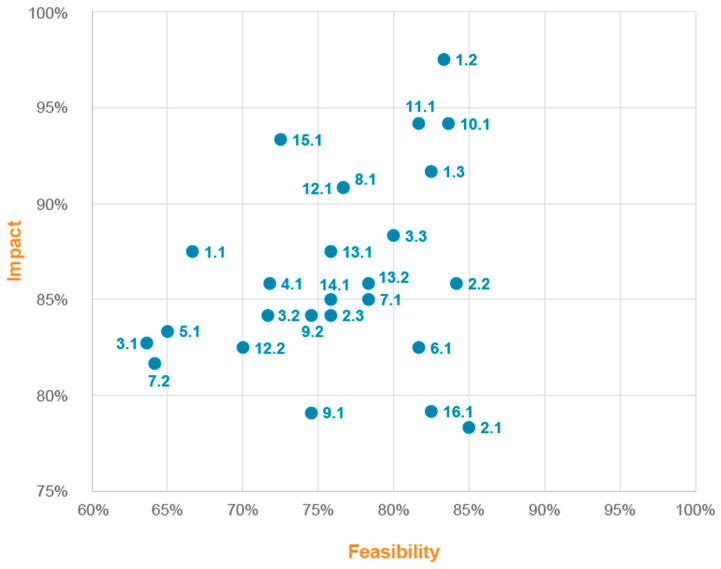
Distribution of improvement proposals identified according to impact and feasibility score. Having established the areas with potential for improvement, the impact and feasibility of the improvement ideas for each improvement opportunity were assessed. For each of the improvement proposals, the main factors hindering their implementation were assessed in terms of economic investment, time, legal considerations, and technological, human, and material resources.

Figure 2 shows the dispersion of ideas according to impact and feasibility score. In-hospital nutritional screening (improvement idea 1.2), participation of the NST on tumour boards (10.1), improved coordination between the oncology department and the NST (11.1), and training in nutritional screening (1.3) scored highest in terms of feasibility/impact, with values of 97.5%, 94.2%, 94.17%, and 91.7% for impact; and feasibility percentages of 83.3%, 83.6%, 81.7%, and 82.5%, respectively. Overall, lack of time and human resources were the primary obstacles found by investigators for these ideas for improvement to be rolled out in clinical practice.

Using a mental mapping methodology, the working group analysed each improvement proposal in terms of what, who, how, and when.

### 3.2. Results of Implementation in Clinical Practice of Proposals for Improvement with the Highest Impact/Feasibility Ratio

**Figure 3:**
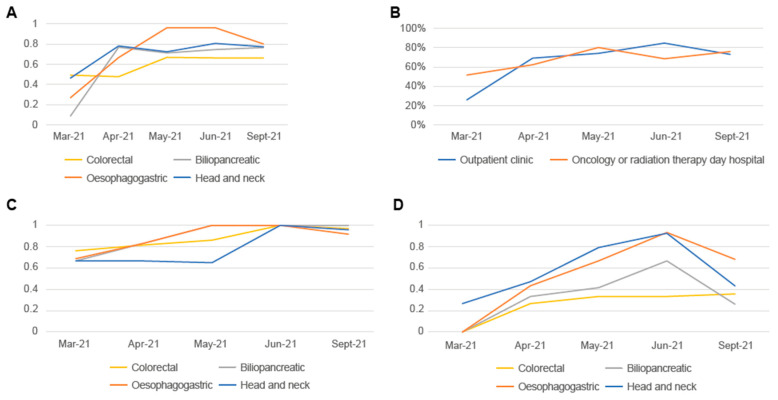
Nutritional assessments. (**A**) Percentage of clinical histories with nutritional assessment results among non-hospitalised patients undergoing nutritional screening by tumour type. (**B**) Percentage of clinical histories with nutritional screening results among non-hospitalised patients in outpatient clinics or oncology/radiation therapy day hospitals. (**C**) Percentage of clinical histories with nutritional assessment results among hospitalised patients undergoing nutritional screening by tumour type. (**D**) Average percentage of tumour board sessions in which a member of the NST participated.

The percentage of oncology clinical histories with nutritional screening results in non-hospitalised patients increased throughout the study for all four tumour types (Figure 3A). Most nutritional screening was performed for patients with oesophagogastric tumours, which approached 100% between May and June, followed by head and neck, colorectal, and biliopancreatic tumours. Between May and June, the percentage of patients screened plateaued, with a slight decrease as of June for oesophagogastric tumours.

The assessment of nutritional screening in non-hospitalised patients according to the unit or department where the screening was performed maintains a similar trend in both outpatient clinics and oncology/radiation therapy day hospitals, reaching approximately 80% of clinical histories (Figure 3B).

In hospitalised patients, however, nutritional screening was higher, reaching 100% as of May for patients with oesophagogastric cancer and patients with biliopancreatic cancer, and as of June, for patients with colorectal or head and neck cancer (Figure 3C). Between June and September, a decrease in screenings was observed, with the mean remaining above 90% in all four tumour types.

There was a general upward trend in the percentage of tumour board sessions involving a member of the NST, with the exception of colorectal tumours, since attendance did not occur at the decision of the medical team in one of the three hospitals in this case (Figure 3D). The highest percentage of participation was observed in head and neck tumours, followed by oesophagogastric, biliopancreatic, and colorectal tumours. In addition, a drastic decrease in general participation was seen from June onwards, except in cases of colorectal tumour in which no changes occurred.

**Figure 4:**
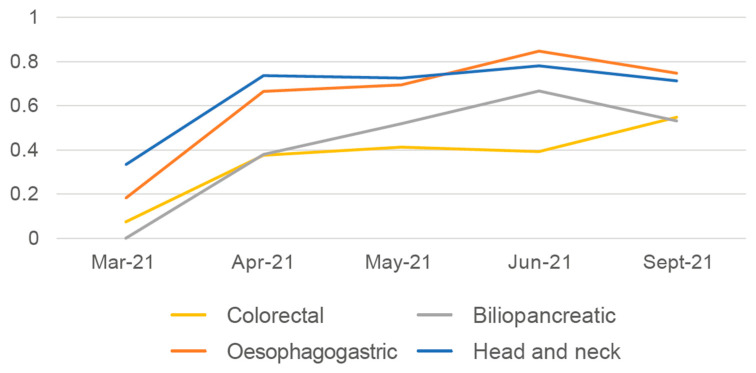
Average percentage of patients assessed by the NST on hospital tumour boards.

Lastly, in the analysis of the percentage of patients evaluated by the NST at the tumour board sessions, an increase was seen between March and April for all four tumour types, which subsequently plateaued (Figure 4). On average, the patients most evaluated at the board sessions were those with head and neck or oesophagogastric tumours, followed by those with biliopancreatic tumours or colorectal tumours.

## 4. Discussion

The NOA project seeks to improve the ways in which malnutrition related to oncological disease are managed by describing current nutritional interventions, their analysis, and their improvement to develop an integrated care plan (oncology and nutrition) with the minimum requirements to be implemented across all healthcare sites. During phases 2 and 3 of the NOA project, proposals were identified, prioritised, described, and evaluated, including, due to their greater impact and feasibility, in-hospital nutritional screening and participation of the NST on tumour boards.

Study participants considered that in-hospital nutritional screenings should be a universal and basic procedure after first contact with the patient and then periodically during treatment and follow-up, as indicated by clinical nutrition guidelines [10,11]. The use of the MUST (Malnutrition Universal Screening Tool) as a basic screening tool was considered, since it is included in the support process of clinical nutrition and dietetics of the Department of Health of the Regional Government of Andalusia [22]; it is also one of the most commonly used tools in clinical practice among the options available [28]. MUST is a quick and simple tool that has been validated as a screening system in adult patients for medical conditions in which nutritional assessment is required, as is the case with oncology [24]. MUST assesses the impairment of functional status, body composition, and physical function, and its score predicts the clinical course of the disease [29].

Another key aspect identified during the project is the involvement of healthcare staff trained in clinical nutrition and dietetics in the care of cancer patients from the time of diagnosis, with the participation of the NST on the tumour board and in coordination with the oncology team during follow-up. Malnutrition may progress to irreversible loss of fat and muscle mass or cancer cachexia [30]. Therefore, prompt identification and treatment are critical. A reduction in weight loss, as well as a stabilisation of body mass index, were seen when patients received early nutritional intervention, whether this was with nutritional counselling or by oral supplementation, which had a positive impact on their quality of life and response to treatment [31].

The aim of this project was to implement in-hospital nutritional screening and the participation of the NST on tumour boards, with monthly and quarterly monitoring by analysing different indicators over a six-month period (March–September 2021). In general, and taking into account the limitations of time and personnel, intensified by the COVID-19 pandemic and the summer holiday season, the measures were generally successfully adopted at the three participating healthcare sites and for all types of tumours analysed, though at different speeds and starting points for each of them. Over the course of the six months, an increase was observed in the percentage of non-hospitalised patients undergoing nutritional screening—a higher percentage in those with oesophagogastric tumours, followed by head and neck, colorectal, and biliopancreatic tumours. In hospitalised patients, the percentage of screening was higher in patients with oesophagogastric tumours, followed by colorectal, biliopancreatic, and finally, head and neck tumours, considering that the number of patients admitted with the latter was lower than the rest (so the population sample of patients with this type of tumour may not be very representative). The participation of a member of the NST on tumour boards has increased over the course of the project, starting in most cases from a baseline situation (March) of low attendance. Participation was greater on the head and neck tumour boards, followed by oesophagogastric, biliopancreatic, and colorectal tumour boards. The percentage of patients assessed by the NST reached values of 100%, with a higher proportion in patients with head and neck tumours and oesophagogastric tumours.

The design of the NOA study is one of its main strengths. Its use of participation and consensus techniques makes it possible to be readily comprehended, shared, and contributed to by investigators, and to efficiently reach a consensus on the proposals. The involvement of healthcare staff from different sites and disciplines provides a variety of perceptions and perspectives, while enriching everyone’s experience. However, all analysis was performed in the autonomous community of Andalusia and in general hospitals, so extrapolating the results to other regions and types of healthcare centres is its main limitation. Moreover, our study was focused on the evaluation of the implementation of improvement points, missing information on the impact of their implementation on patient outcomes. Future observational studies will evaluate the impact of the proposed plan in long-term routine clinical practice.

## 5. Conclusions

The NOA study proposes a prototype for an integrated nutritional oncology care plan and establishes key improvement points for each phase of the plan, demonstrating its direct impact on nutritional intervention and establishing the basis for future studies in other regions or contexts and focused on health outcomes (not only on process indicators). The results of the NOA project, in line with the regional and national cancer plans, as well as with the objectives of Europe’s Beating Cancer Plan [12], demonstrate that multidisciplinary work should be the working model among the medical specialities involved in the treatment of cancer patients.

## Figures and Tables

**Table 1:** Proposed improvement ideas.

Opportunity for Improvement		Description of the Improvement Idea		Obstacles	Phase
1	No nutritional screening by family doctor, specialists, or oncologists	1.1	Nutritional screening in primary care	Time and human resources	Pre-diagnosis, diagnosis
		1.2	In-hospital nutritional screening	Time and human resources	Pre-diagnosis, diagnosis, treatment
		1.3	Nutritional screening training	Time and human resources	Diagnosis, treatment, monitoring, and follow-up
2	Inadequate documentation, nutritional information, and existing resources and support services for patients and relatives	2.1	Patient information materials	Necessary materials, technology, time, and economic investment	Treatment, monitoring, and follow-up
		2.2	Basic nutritional counselling	Time and human resources	Pre-diagnosis, diagnosis, treatment, monitoring, and follow-up
		2.3	Advanced nutritional counselling	Time and human resources	Diagnosis, treatment, monitoring, and follow-up
3	Lack of nutritional training for patients and caregivers	3.1	Unreliable nutrition information resource alert system	Technology, time, and legal aspects	Pre-diagnosis, diagnosis, treatment, monitoring, and follow-up
		3.2	Nutritional training for patients and relatives	Time and human resources	Diagnosis
		3.3	Follow-up of patient nutritional status	Time and human resources	Treatment, monitoring, and follow-up
4	Inadequate involvement of family doctors in the nutrition of cancer patients	4.1	Improve training of primary care professionals on nutrition in cancer patients	Time and human resources	Pre-diagnosis
5	Inadequate cancer-specific psychological support for cancer patients and relatives, related to nutrition	5.1	Enhance cancer-specific psychological support in relation to nutritional status	Human resources	
6	Excessive paperwork for nutritional supplements	6.1	Simplify prescription procedures for nutritional supplements	Legal aspects	Diagnosis, treatment
7	Inadequate financial and social support for nutrition for patients and caregivers	7.1	Review the indications of HEN covered by the NHS	Legal aspects and human resources	Monitoring and follow-up
		7.2	Referrals to social worker as food and nutrition support in cancer patients	Economic investment and human resources	
8	Inadequate nutritional assessment and patient preparation prior to radiation therapy and chemotherapy	8.1	Improve nutritional assessment and preparation prior to cancer treatment	Time and human resources	Pre-diagnosis, diagnosis
9	Inadequate pre-habilitation before surgery (with major variability between centres)	9.1	Increase the surgical and anaesthesia teams’ understanding of nutrition	Time and human resources	Diagnosis, treatment
		9.2	Incorporate coadjuvant nutritional measures before cancer surgery	Time, economic investment, and human resources	
10	Non-participation of the NST on and lack of nutritional protocols for the tumour board.	10.1	NST participation on hospital tumour boards	Time and human resources	Diagnosis, treatment
11	Improve protocol for referral to NST	11.1	Improve coordination between the oncology department and the NST	Time and human resources	Diagnosis, treatment, monitoring, and follow-up
12	Inadequate access and communication between the NST and professionals, PCU and home hospitalisation	12.1	Improve coordination between PCU, home hospitalisation and NST	Time and human resources	Monitoring and follow-up
		12.2	Implement telemedicine in all its versions	Economic investment and technological resources	Treatment, monitoring, and follow-up
13	Inadequate specific nutrition consultations during treatment (in-person or telephone) for patients	13.1	Raise awareness of nutrition	Time and human resources	Pre-diagnosis, diagnosis, treatment, monitoring and follow-up
		13.2	Improve accessibility and care provision in the NST	Time, technology, and human resources	Pre-diagnosis
14	More accessible clinical history	14.1	Specific section on nutrition in the electronic clinical history of the DIRAYA	Technology	Pre-diagnosis, diagnosis, treatment, monitoring, and follow-up
15	Lack of exercise and rehabilitation programs	15.1	Prescription of exercise as coadjuvant to nutrition	Economic investment and human resources	Diagnosis, treatment, monitoring, and follow-up
16	Improved organoleptic characteristics of preparations and supplements	16.1	Different nutritional supplement forms	Economic investment and technological resources	Monitoring and follow-up
17	Parenteral nutrition.	17.1	Parenteral nutrition support	Not evaluated	
18	Enteral nutrition.	18.1	Nutritional support via the enteral route	Not evaluated	Diagnosis, treatment

DIRAYA: Andalusian eHealth Strategy and system; HEN: home enteral nutrition; NHS: National Health System; NST: Nutritional Support Team.

## Data Availability

The data presented in this study are available on request from the corresponding author.

## Supplementary Materials

The following supporting information can be downloaded at: https://www.mdpi.com/article/10.3390/nu15020292/s1, Table S1: Study participants, site and speciality.
