# Supplementary material for: Evaluation of Nutritional Interventions in the Care Plan for Cancer Patients: The NOA Project
Source: Nutrients. 2023 Jan 6;15(2):292. doi: 10.3390/nu15020292 (PMC9866236; doi:10.3390/nu15020292)
Supplement: Supplementary file 1 [file nutrients-15-00292-s001.zip › nutrients-2125331-supplementary.pdf]

Supplementary Table S1. Study participants, site and speciality.

Name	Site	Speciality
	**Phase 2**	
Pedro Pablo García Luna [a]	Hospital Universitario Virgen del Rocío (Seville)	Nutrition
Gabriel Olveira	Hospital Regional Universitario (Malaga)	Nutrition
David Vicente Baz [b]	Hospital Universitario Virgen de la Macarena (Seville)	Oncology
Alfonso Calañas Continente	Hospital Reina Sofía (Cordoba)	Nutrition
Juana María Rabat Restrepo	Nutrition	Nutrition
María Dolores Mediano Rambla	Hospital Universitario Virgen de la Macarena (Seville)	Oncology
Laura Díaz Gomez	Hospital de Jerez de la Frontera (Cadiz).	Oncology
Teresa Muñoz Lucero	Hospital Puerta del Mar (Cadiz)	Oncology
Isabel Castanedo Córdova	Hospital Universitario Virgen de la Victoria (Malaga)	Oncology
Carmen Pilar Jiménez Lorente	Virgen del Mar Health Centre (Almeria)	Nutrition
Ana María Villarubia Pozo	Hospital Universitario Virgen del Rocío (Seville)	Nutrition
Teresa Brozeta Benítez	Hospital Universitario Virgen del Rocío (Seville)	Nutrition
Javier Salvador Bofill	Hospital Universitario Virgen del Rocío (Seville)	Oncology
	**Phase 3**	
Gabriel Olveira	Hospital Regional Universitario (Malaga)	Nutrition
Francisco Sánchez Torralvo	Hospital Regional Universitario (Malaga)	Nutrition
Antonio Rueda	Hospital Regional Universitario (Malaga)	Oncology
Marta Muñoz Ayllon	Hospital Regional Universitario (Malaga)	Oncology
Pedro Pablo García Luna	Hospital Universitario Virgen del Rocío (Seville)	Nutrition
Pablo Remon Ruiz	Hospital Universitario Virgen del Rocío (Seville)	Nutrition
Juana Rabat	Hospital Universitario Virgen de la Macarena (Seville)	Nutrition
Juan Jesús García González	Hospital Universitario Virgen de la Macarena (Seville)	Nutrition
Javier Salvador Bofill	Hospital Universitario Virgen del Rocío (Seville)	Oncology
Jerónimo Pachón	Hospital Universitario Virgen del Rocío (Seville)	Radiation Oncology
Milagros de la Calle	Hospital Universitario Virgen del Rocío (Seville)	Oncology
David Vicente	Hospital Universitario Virgen de la Macarena (Seville)	Oncology
Teresa García Manrique	Hospital Universitario Virgen de la Macarena (Seville)	Oncology

[a]: Project Director and Principal Investigator. B: Oncology Coordinator and Principal Co-Investigator.
